# SwiftLib: rapid degenerate-codon-library optimization through dynamic programming

**DOI:** 10.1093/nar/gku1323

**Published:** 2014-12-24

**Authors:** Timothy M. Jacobs, Hayretin Yumerefendi, Brian Kuhlman, Andrew Leaver-Fay

**Affiliations:** 1 Department of Biochemistry, University of North Carolina at Chapel Hill, Chapel Hill, NC 27599, USA; 2 Lineberger Comprehensive Cancer Center, University of North Carolina at Chapel Hill, Chapel Hill, NC 27599, USA

## Abstract

Degenerate codon (DC) libraries efficiently address the experimental library-size limitations of directed evolution by focusing diversity toward the positions and toward the amino acids (AAs) that are most likely to generate hits; however, manually constructing DC libraries is challenging, error prone and time consuming. This paper provides a dynamic programming solution to the task of finding the best DCs while keeping the size of the library beneath some given limit, improving on the existing integer-linear programming formulation. It then extends the algorithm to consider multiple DCs at each position, a heretofore unsolved problem, while adhering to a constraint on the number of primers needed to synthesize the library. In the two library-design problems examined here, the use of multiple DCs produces libraries that very nearly cover the set of desired AAs while still staying within the experimental size limits. Surprisingly, the algorithm is able to find near-perfect libraries where the ratio of amino-acid sequences to nucleic-acid sequences approaches 1; it effectively side-steps the degeneracy of the genetic code. Our algorithm is freely available through our web server and solves most design problems in about a second.

## INTRODUCTION

*In vitro* evolution couples genetic diversity generation with either a screen or a selection to identify proteins with some desired phenotype. Although creating highly diverse DNA libraries is trivial, the efficient isolation of the sought-after phenotype presents a bottleneck, limiting the number of DNA sequences that can be tested. Techniques for selecting crossover loci in gene shuffling (1–3), for open-reading-frame selection (4,5), for screening neutral drift libraries (6) and for screening restricted-alphabet libraries (7–9) all aim to distill sequence space to a manageable size while maximizing the likelihood that the remaining sequences will yield the desired phenotype.

Degenerate codon (DC) libraries are attractive in that they focus diversity to regions the library designer thinks will be most productive, they can be molded to include as much or as little diversity at particular positions as is necessary, and they are relatively inexpensive to make. Unfortunately, canonical ‘NNN’ diversification (N = A, C, G or T) can be used at only a small number of positions before exceeding the experimental size limits. Take for example the 10^7^ diversity limit imposed by yeast surface display, a rather middle-of-the road limit; >10^3^ limit for 96-well format screening and <10^13^ limit for mRNA display. NNN diversification would exceed a 10^7^ diversity limit if used at a mere four positions. NNN is particularly inefficient since it commits 64 DNA sequences to produce only 20 distinct amino acid (AA) sequences. ‘NNK’ (*K* = G or T) is better, since it uses only 32 DNA sequences for the same 20 AAs. There are more tricks to increase the AA:DNA ratio: the ‘22c trick’ (10) uses three separate DCs to get all 20 AAs from only 22 DNA sequences, and the ‘small intelligent libraries’ technique (11) uses four DCs to get all 20 AAs for exactly 20 DNA sequences. Still, using 20 DNA sequences allows full randomization at only five positions before bumping into a 10^7^ diversity limit. To randomize more positions requires limiting the AA diversity. The NDT DC (D = A, G or T), which codes for 12 AAs using 12 DNA sequences has been suggested as one way to reduce the AA diversity (9). Multiple-sequence alignments (12) or computational protein design (13) have also been used to suggest which AAs might be worth considering at each position. However, having a set of candidate AAs at each position leaves the library designer with deciding which DCs to use so that diversity is spread out most productively while adhering to the diversity limit. This paper presents an efficient algorithm for making this decision.

Early work in automating DC optimization mostly focused on matching some target AA distribution by creating ‘spiked’ DCs, where the nucleotide ratios are not uniform (14–20). These efforts focused on single positions at a time and made no attempt at trading off between positions. Firth, Patrick and Blackburn created and still host a web server for selecting DCs for a given set of AAs (21,22) which is a significant boon to anyone looking to partially automate the process of designing a DC library.

Mena and Daugherty introduced *LibDesign*, the first algorithm we are aware of for whole library optimization (23). It takes as input a set of sequences for the positions to be randomized, taken either from protein design trajectories or from multiple sequence alignments and tallies the AA frequencies at each position. Starting with the (2^4^ − 1)^3^ = 3375 possible DCs (2^4^ representing the number of bit strings of length 4 to give all combinations of A, C, G and T; the −1 to throw out the bit string where all four nucleotides are absent), LibDesign chooses small subsets (6 or 7 DCs) for each position by choosing first the smallest DC that gives the most commonly observed AA (really, just a single codon), then the smallest DC that gives the two most commonly observed AAs and so on, until it has a DC that covers all the observed AAs. It then uses brute-force enumeration of all codons in the subsets, looking for combinations with high scores (more on this score later) and that stay below a specified diversity limit.

Treynor *et al*. (24) introduced a technique for building DC libraries while considering energies computed by protein design software, treating the library optimization problem (‘what DC should be assigned to each position?’) as a variant of the rotamer optimization problem (‘what rotamer should be assigned to each position?’) and using previously developed dead-end elimination theorems to solve it. (Briefly, rotamers or rotational isomers, represent discrete side-chain conformations that differ from each other only in their dihedral angles (25).) Instead of looking at rotamer-pair energies, their optimization algorithm looks at the average AA pair energies for a given pair of DCs; each AA pair energy is taken simply as the rotamer interaction energy between the two rotamers that interact most favorably with the template structure (and sequence). One obvious drawback of this technique is that it does not consider how the rotamers of two AAs might relax in each other's presence. It also lacks a way to limit the size of the resulting library and must be run repeatedly (requiring the user to exclude AAs that might be responsible for producing too much diversity) until a library of the right size is produced.

Allen *et al*. (26) followed with another library design technique, CLEARSS. It begins by computing for every position and for every AA-subset size the optimal AA subset as defined by an input set of scored sequences (e.g. the 1000 lowest-energy sequences that emerged from a set of protein design trajectories). It then enumerates all assignments of per-position sizes so that the product of those sizes is within a user-specified size range and picks the best assignment. The algorithm's reliance on brute-force enumeration means that it is quite slow. It can be made to run quickly when the number of AAs at each position is either very low or very high so that there is little to enumerate, but it is much slower when an intermediate number of AAs is used. Curiously, the authors designed their algorithm to enumerate based on the number of AA sequences instead of the number of DNA sequences, which represents the actual experimental constraint. They also incorrectly assert that the optimal set of AAs for a particular position will be no larger than the set that contains all of the AAs that appear in the input designs; this would not be true if a DC pulled in AAs in addition to the desired ones, as DCs frequently do. Based on this assertion, CLEARSS restricts the number of AA subsets it examines and as a result may miss the optimal DC choice in spite of the fact that it uses brute force enumeration.

Parker *et al*. (27) introduced both a dynamic programming (DP) solution and an integer-linear programming (ILP) solution to solve a DC library optimization problem where the library is guided by sequence alignments only and the designer has not even chosen which positions to randomize. They make the very relevant observation that trying to optimize the library so that pairwise information is included (e.g. including PHE at residue 10 only if ARG is included at position 20 because PHE10 is only ever seen if ARG20 is also present) makes the library optimization problem NP-Complete; thus the need for an ILP solution to optimize their pairwise quality and novelty metrics. The DP algorithm they give is quite different from the one presented here; notably, it is unable to enforce a limit on the size of the resulting library, merely a limit on the number of positions which are randomized. Their ILP solution, in contrast, is able to enforce a library size limit. Chen *et al*. (28) extended this result in designing a DC library with ILP where multiple DCs were allowed at a single position, with the restriction that only one position among those that would be covered by the same primer—those that lie in a single *stretch* of DNA—be allowed to use multiple DCs.

The DP algorithm presented here also allows multiple DCs per position, but removes the restriction that only one position per stretch use multiple DCs. Experimentally, the construction of such a library would require purchasing multiple primers to cover the stretch, where the number of primers needed is the product of the number of DCs used at each position within the stretch. The algorithm allows the user to limit the total number of primers that could be used and, with that limit, determines the optimal way to distribute the use of those primers. Surprisingly, the use of multiple DCs allows the algorithm to come up with very efficient libraries where the degeneracy of the genetic code can all but be avoided so that, at least for the test cases examined here, the AA:DNA ratio approaches 1. The implementation that we have deployed online, which we call *SwiftLib*, offers an accessible alternative to the previously published ILP approaches (27,28) because it requires no back end. SwiftLib is implemented in only ∼2K lines of JavaScript and runs inside web browsers; the code executes on the user's computer. It is, to our knowledge, the first web server for optimizing DC libraries. SwiftLib is accessible at http://rosettadesign.med.unc.edu/SwiftLib.

## MATERIALS AND METHODS

### DP for one degenerate codon

We have formulated the optimization of DC libraries with a linearly-additive error function that we wish to minimize, subject to the constraint that the library size not exceed a given limit. This error function treats each residue separately, omitting any consideration of residue-pair information that might be present. Because it is linearly additive and each of its errors are integers, its optimization admits a rapid DP solution.

The input for the problem is a 20 × *n* table of AA counts for *n* designable residues and a diversity limit, *L*. The count *C*_*i*_(*a*) is the number of times that AA *a* ∈ *A* appeared in the set of input sequences at position *i*, where *A* is the set of AA. We define the error for choosing a particular DC $d \in \mathbb {D}$ at position *i* as

(1) \begin{equation*} E_i = \sum _{a \in A} \delta (a \not\in p(d)) C_i(a) \end{equation*}

where *p*(*d*) is the set of AAs produced by *d*, and $\delta (x \not\in y)$ is the delta function which yields a value of one if *x* is absent from the set *y* and zero otherwise and $\mathbb {D}$ is the set of all 3375 DCs. Effectively, this objective function penalizes the exclusion of AAs that were observed in the input sequences, with greater penalties applied to AAs that appeared the most. The error generated by an assignment *D* = {*d*_1_…*d*_*n*_} to all *n* designable positions is simply the sum of the individual errors:

(2) \begin{equation*} E(D) = \sum _i^n E_i( d_i ). \end{equation*}

Let |*d*| represent the number of DNA sequences defined by a particular DC *d* and |*D*| represent the product $\prod\nolimits_i^n {|d_i |}$. Then the optimization problem can be formulated as:

\begin{equation*} \min _{D \in \mathbb {D}^n }\ E(D) \end{equation*}

subject to the constraint that |*D*| ≤ *L*.

This problem can be inverted so that instead we solve for the smallest library that achieves any particular error level *e*. If we have a list of these library sizes for all possible errors, then we can simply pick out the smallest error that can be generated with a library beneath the given limit. This optimization strategy mirrors the one that Bellman originally described when inventing DP (29). Let $s_i^e$ denote the smallest size *min*|*d*| of all DCs that at position *i* generate a particular error level *E*_*i*_(*d*) = *e* and infinity if there are no DCs that produce that error level. Let $S_{i-1}^e$ denote the size of the smallest sub-library *D*_*i* − 1_ = {*d*_1_…*d*_*i* − 1_} defined over the range between the first designable position up to and including position *i* − 1 that generates error *E*(*D*_*i* − 1_) = *e*. $S_{i}^e$ can be expressed recursively as:

(3) \begin{equation*} S_{i}^e = \min _{0 < e^{\prime } \le e } S_{i-1}^{e-e^{\prime }} \times s_{i}^{e^{\prime }} \end{equation*}

with the recursion bottoming out at $S_1^e = s_1^e$. Since the error is integral, we can turn this recursion on its head and build up a table of partial solutions. If the maximum error that can be produced at any position is *m*, then two *n* × *nm* tables are needed to hold the solution. The DP algorithm is as follows: For all 1 < *i* ≤ *n* and for all 0 ≤ *e* ≤ *mi*, compute

(4) \begin{equation*} S[ i, e ]{} = \min _{0 \le e^{\prime } \le e^*} \ \ S[ i-1, e-e^{\prime } ] \times s_i[ e^{\prime } ] \end{equation*}

and

(5) \begin{equation*} T[ i, e ] {} = \mathop {{\rm arg}\,\min }\limits_{0 \le e' \le e^* } \ \ S[ i-1, e-e^{\prime } ] \times s_i[ e^{\prime } ]. \end{equation*}

where *e*′ represents the choice of error contributed by position i, e* is the smaller of *e* and *m* and *T* represents a traceback table that can be used to reconstruct the optimal degenerate-codon assignment after the smallest error that satisfies the library size limit has been identified. This algorithm populates the two tables in O(*n*^2^*m*^2^) time and the traceback to reconstruct the optimal solution takes O(*n*) time.

This solution can be trivially extended to place penalties on AAs, including the STOP codon, that the user would prefer to exclude (but not forbid) from the library, as long as those penalties are also integral. It is also possible to forbid or to require AAs by restricting the set of DCs from which to chose; this is more of a preprocessing addition than it is a modification to the DP algorithm.

### DP for multiple degenerate codons

The DP algorithm can also be extended to allow multiple DCs at a single position while constraining the total number of primers that must be purchased to accommodate the extras. Using multiple DCs at a single position allows the exploration of a wider set of AAs while keeping the size of the library down; it is cheaper (in terms of library size) to get the AA set {C,W,Y} using the two DCs TRT (R = A or G) and TGG, with a DNA size of 3 than it is to use the single DC TRK (K = G or T) with a DNA size of 4 and this economy is especially important since TRK also pulls in a STOP codon. Indeed, if the library-designer wishes to forbid STOP codons entirely, then the AA set {C,W,Y} can only be designed if multiple DCs are considered. However, it is more expensive (in terms of money) to use multiple DCs because more primers have to be purchased.

We assume that the primer boundaries are defined ahead of time; this allows us to talk about a *stretch* of DNA that contains a set of designable positions. (We do not consider here the more challenging problem of trying to simultaneously optimize the DCs and the stretch boundaries, which would require knowledge of the G/C content of the DNA between the designable positions and the annealing temperature for the polymerase chain reaction (PCR) used for gene assembly.) For a single stretch, the number of primers that must be purchased is the product of the number of DCs chosen at each of its randomized positions; the cartesian product of the selected DCs must be purchased to cover all combinations.

The user may wish to constrain this problem by defining limits on the number of DCs per position, *L*_p_, on the number of primers to order per stretch, *L*_s_ and on the number of primers to purchase total, *L*_T_. In the analysis that follows, we consider the sensical assignment of values 1 ≤ *L*_p_ ≤ *L*_s_ ≤ *L*_T_ only, and also assume that *L*_T_ is at least as large as the number of stretches.

The DP solution to this problem is similar to the one given above; it again solves for the smallest library that produces a given error, but also pays attention to the primer counts: at iteration *i*, DP solves for the smallest library containing all positions up to and including position *i* given that it produces an error level *e*, using *j* primers total and using *k* primers to cover *i*'s stretch. Now, the number of extra primers required if *j*′ DCs are used at position *i* depends on how many codons have already been used at all the other positions on the same stretch. If *k*′ represents the product of the number of DCs at all positions less than *i* that are on the same stretch as *i*, then using *j*′ DCs at position *i* means using *j*′ × *k*′ primers for that stretch; if *j*″ represents the number of primers used total for all positions less than *i*, then using *j*′ DCs at position *i* will have one of two possible effects on the total number of primers demanded, depending on whether or not position *i* is the first position in a stretch: if *i* is the first position in a stretch, it will mean using *j*″ + *j*′ primers total and if *i* is not the first position in a stretch, it will mean using $j^{\prime \prime } + \frac{j^{\prime }-1}{j^{\prime }}{k^{\prime }}$ primers total.

The DP solution for this problem is given by the following two equations:

(6) \begin{eqnarray*} S[ i, j, j^{\prime }, e ] &= \mathop {\min }\limits_{1 \le k' \le L_s } \ \ \mathop {\min }\limits_{0 \le e' \le e^* } \nonumber \\ & S[ i-1, j - j^{\prime }, k^{\prime }, e-e^{\prime } ] \times s_{i}[ j^{\prime }, e^{\prime } ] \end{eqnarray*}

if position *i* is the first randomized position for a stretch and

(7) \begin{eqnarray*} S[ i, j, k, e ] = {} & \mathop {\min }\limits _{1 \le j^{\prime } \le j^* \ \mid \ k/j^{\prime } \in \mathbb {I} } \ \ \ \ \ \mathop {\min }\limits _{0 \le e^{\prime } \le e^*} \nonumber \\ & S[ i-1, j-\frac{j^{\prime }-1}{j^{\prime }}k, \frac{k}{j^{\prime }}, e-e^{\prime } ] \times s_{i}[ j^{\prime }, e^{\prime } ] \end{eqnarray*}

if position *i* is not the first randomized position for a stretch. where j* is the smaller of *j* and *L*_*p*_. The table *s*_*i*_ holds the smallest-library sizes for position *i* for each error value between 0 and *m*, and for each number of DCs between 1 and *L*_p_. The equations for the traceback table are similarly constructed.

This DP algorithm runs in ${\rm O}( n^2m^2L_{\rm s}^2L_{\rm T} )$ time plus an initial expense of computing the *s*_*i*_ tables, which requires ${\rm O}(n3375^{L_{\rm p}})$ time to consider all combinations DCs. This initial expense can be shaved by first computing the subset of DCs that produces the smallest error for each distinct coverage of AAs that contribute to the error (either by their absence or their presence) and then enumerating combinations of DCs from this subset. The speedup offered by this technique decreases as more and more AAs contribute to the error. In our test cases, the sizes of these subsets are in the range of 100–400, making the initial expense of populating the *s*_*i*_ tables negligible. This algorithm requires O(*n*^2^*mL*_s_*L*_T_) memory to store the *S* and *T* tables.

In both DP solutions, a very simple speedup can be obtained if one is only interested in knowing about the minimum-error library or about the *N* lowest-error libraries with sizes less than the given diversity limit: if the total library error is iterated over in the outermost loop (instead of the position), one may stop as soon as the first library of size less than *L* is found or as soon as the first *N* libraries of size less than *L* are found. This output-sensitive algorithm runs in ${\rm O}( n(k+1)^2L_{\rm s}^2L_{\rm T} )$ time, where *k* is the total error for the worst library sought (i.e. the smallest total error if only one library is sought). For this reason, SwiftLib runs fastest when it is able to find a low-error solution. Furthermore, the use of a sparse array can reduce the memory overhead significantly; since SwifLib is implemented in JavaScript, the JavaScript interpreter has this option.

## RESULTS

### Library designs

We chose two library design test cases where manual solutions had been created before our DP algorithm was implemented. In both cases, Rosetta (30–32) was used to simultaneously redesign several positions, and these redesigns were used to guide the manually selected DCs. or each test case, we used DP to design three DC libraries with three different library design goals: (i) allowing only one DC per position, (ii) allowing at most two DCs per position allowing twice as many primers as there are stretches and (iii) allowing at most three DCs per position and increasing the limit on the total number of primers. The libraries were assessed only on how well they capture the sought-after sequence diversity; the DP libraries have not been synthesized or screened.

We compared the results from DP to the previously published LibDesign algorithm (23). This algorithm is a natural comparison for ours as its inputs and goals are nearly identical. LibDesign takes a set of input sequences, selects a small set of DCs for each position based on the AA counts from the input sequences and then enumerates all combinations of DCs. The LibDesign score for a library is the number of sequences in the input set that are generated by the library: every AA in a particular sequence has to be encoded in the library in order for that sequence to contribute to the score. LibDesign attempts to maximize its score (in contrast to SwiftLib which minimizes its error metric). This score is at the furthest end of the spectrum from our error metric in complexity: our error term treats each position independently, Parker *et al*.'s ILP alogirthm is more complex since it considers residue-pair information, but LibDesign captures higher-order inter-residue dependencies still.

The AA counts for Problem 1 are given in Table 1. These counts came from 200 protein design trajectories at nine positions in a particular protein. The designed libraries are given in Table 2. The manual solution was produced by a novice library designer who focused only on the most-commonly observed AAs at each of the designable positions and sought out DCs that were capable of covering these AAs. For this reason, the library contains much higher per-position error than the DP libraries. For this problem, the third DP solution was constrained to use 15 or fewer primers.

**Table 1.** Problem 1: Rosetta (30–32) was used to generate 200 designs for a set of surface residues in a protein-interface-design application using the 1XBI PDB

pos	268	269	270	271	272	276	330	331	332
A	3	8	1	81	4	105	10	2	1
C	0	0	0	0	0	0	0	0	0
D	8	5	0	0	0	0	29	7	9
E	23	7	0	0	0	0	21	23	17
F	0	0	0	0	0	0	0	0	0
G	0	0	0	1	0	59	4	1	5
H	1	0	0	0	0	0	0	1	9
I	10	2	98	0	0	0	0	0	1
K	22	0	0	0	0	0	5	4	9
L	17	8	5	0	0	0	0	14	0
M	13	9	5	0	2	0	3	7	4
N	6	4	0	0	0	0	8	19	15
P	0	0	0	0	0	0	0	0	0
Q	42	2	0	0	1	0	6	29	18
R	35	4	0	0	0	0	27	12	21
S	7	44	8	113	43	36	45	34	29
T	6	58	23	4	30	0	42	46	57
V	7	49	60	1	120	0	0	0	5
W	0	0	0	0	0	0	0	1	0
Y	0	0	0	0	0	0	0	0	0

These are the AA counts for each of the nine designed positions. The designable positions are contained in two stretches, divided by the vertical bar.

**Table 2.** Solutions to library design problem 1: the manual solution to the first library design problem, along with the best solution produced by LibDesign (23) and three solutions produced by DP

Solution	pos	268	269	270	271	272	276	330	331	332	Totals
Manual	DCs	VDR	RBT	RYY	KCT	RBT	RVT	RVW	VNW	VVW	105^a^
	AAs	EGIKLM	AGIS	AITV	AS	AGIS	ADGN	ADEGKN	ADEGHIKL	ADEGHK	
		QRV	TV			TV	ST	RST	NPQRSTV	NPQRST	
	#NAs	18	6	8	2	6	6	12	24	18	3.2 × 10^8^
	#AAs	9	6	4	2	6	6	9	15	12	2.5 × 10^7^
	%Des	89	83	100	100	67	50	100	75	89	16.5
	Error	29	39	18	6	3	0	9	8	10	122

LibDesign	DCs	VNK	DYG	RYA	KCA	DYG	KSA	VVK	VNK	VNK	75^a^
	AAs	ADEGHIKLM	ALM	AITV	AS	ALM	AGS	ADEGHKN	ADEGHIKL	ADEGHIKL	
		NPQRSTV	STV			STV	*STOP*	PQRST	MNPQRSTV	MNPQRSTV	
	#NAs	24	6	4	2	6	4	18	24	24	2.9 × 10^8^
	#AAs	16	6	4	2	6	3	12	16	16	5.7 × 10^7^
	%Des	83	100	100	100	83	75	83	79	83	28.6
	Error	0	24	18	6	1	0	3	1	0	53

DP Sol. 1	DCs	VNS	DYG	DYA	KCA	DYG	RSC	RVM	VNS	VNS	157^a^
	AAs	ADEGHIKL	ALM	AIL	AS	ALM	AG	ADEG	ADEGHIKL	ADEGHIKL	
		MNPQRSTV	STV	STV		STV	ST	KNRST	MNPQRSTV	MNPQRSTV	
	#NAs	24	6	6	2	6	4	12	24	24	2.9 × 10^8^
	#AAs	16	6	4	2	6	4	12	16	16	6.4 × 10^7^
	%Des	83	100	100	100	83	75	100	79	83	34.4
	Error	0	24	5	6	1	0	9	1	0	46

DP Sol. 2	DCs	VNS	DBG, RAM	DYA	KCA	DYG	RSC	RVM	VAM, WBG	VNS	173^a^
	AAs	ADEGHIKL	ADEGKL	AIL	AS	ALM	AG	ADEG	DEHKLM	ADEGHIKL	
		MNPQRSTV	MNRSTVW	STV		STV	ST	KNRST	NQRSTW	MNPQRSTV	
	#NAs	24	13	6	2	6	4	12	12	24	2.9 × 10^8^
	#AAs	16	13	6	2	6	4	9	12	16	1.0 × 10^8^
	%Des	83	77	100	100	83	75	100	100	83	33.4
	Error	0	4	5	6	1	0	9	3	0	28

DP Sol. 3	DCs	MKC, RYG, VAM	AKS, RMC, SWA	DYA	DCA	DYG	RSC	ADG, RVC, SAA	DBG, VAM	VNS	192^a^
	AAs	ADEHIKLM	ADEILM	AIL	AST	ALM	AGST	ADEGKM	ADEGHKLM	ADEGHIKLM	
		NQRSTV	NQRSTV	STV		STV		NQRST	NQRSTVW	NPQRSTV	
	#NAs	14	12	6	3	6	4	11	15	24	2.9 × 10^8^
	#AAs	14	12	6	3	6	4	11	15	16	1.9 × 10^8^
	%Des	100	100	100	100	83	75	100	93	83	48.6
	Error	0	0	5	2	1	0	0	0	0	8

The table reports the chosen DCs at each position, the AAs produced by those DCs, the number of nucleic-acid sequences the DCs prescribe (#NA), the number of unique AAs and AA sequences the DCs produce (#AA), the percentage of codons that produce desired AAs and the percentage of library members that contain only desired AAs (%Des), the error as calculated from Equations 1 and 2 and the LibDesign score^a^. DP solution 1 used one DC per position, solution 2 considered two DCs per position with a limit of 4 primers total and solution 3 considered three DCs per position with a limit of 15 primers total. The automated solutions were restricted to a diversity limit of 3.2 × 10^8^, the size of the manually designed library. The maximum achievable LibDesign score for this problem is 200 (higher is better). The manual solution took several hours. LibDesign took 18 min 51 s. The three dynamic-programming solutions took 0.15, 0.28 and 13.57 s.

The DP solutions to Problem 1 have much lower errors than the manual solution. DP solution 1, which like the manual solution uses only a single DC per position, achieves a considerably lower error. It also produces a library with twice as much AA diversity (the number of unique protein sequences), while actually decreasing the DNA diversity. DP solution 2, using at most four primers and two DCs, decreases the error still further from DP solution 1, choosing to use two DCs at positions 269 and 331, where both the manual solution and DP solution 1 had the highest per-position error and a low AA:DNA ratio. It also delivers four times the AA diversity as the manual solution. DP solution 3 chooses to divide its 15 primers into one group of 9, using three DCs for both positions 268 and 269 and one group of 6, using three DCs for position 330 and two DCs for position 331. As a result, its error drops to 8 while its AA:DNA ratio increases to 2:3; at only a single position does it use more than one codon for an AA. It delivers nearly 10× the AA diversity as the manual solution. The fraction of AA sequences also increases as the number of DCs increases so that nearly half of all sequences in DP solution 3 contain only desired AAs, in contrast to one-sixth in the manually constructed library. The DP algorithm was much faster than the manual process; the manual solution took hours to generate but DP solutions 1, 2 and 3 took 0.15, 0.28 and 13.57 s.

The AA counts for Problem 2 are given in Table 3. These counts came from 1000 protein design trajectories at 12 positions in a particular protein. The designed libraries are given in Table 4. The manual solution came from an expert library designer and took advantage of two techniques: the ISOR technique for ensuring that the native AA is present in the library (33) and the use of multiple DCs. Briefly, ISOR ensures that the WT AAs are present in the library by creating extra ‘primers’ by partial digestion of the WT gene. The designer of this library made two mistakes. First, the library's intended size was 10^9^, but it came out more than 10× smaller at just under 10^8^. Second, the library designer meant to use a codon for leucine (‘CTG’) at position 520, but mistakenly chose (and subsequently ordered) the codon ‘CAG’, which codes for glutamine, instead. The error of 61 reported in Table 3 for the manual library was calculated as if ‘CTG’ had been ordered.×

**Table 3.** Problem 2: Rosetta was used to generate 1000 sequences on residues near the Jα helix of the *Avena sativa* LOV2 domain (PDBid 2V0U)

	413	475	477	479	493	495	514	520	528	529	531	532
A	990	3	1	21	323	1	0	0	934	115	0	992
C	0	0	0	0	0	0	0	0	0	0	0	0
D	0	0	0	0	0	0	0	0	0	0	0	0
E	0	**0**	0	0	0	0	0	0	0	0	0	0
F	1	0	460	0	0	964	3	0	0	0	0	0
G	9	0	390	349	351	23	45	0	**17**	5	0	0
H	0	0	34	0	257	**12**	2	0	0	498	0	0
I	0	0	1	630	2	0	86	0	0	0	0	**1**
K	**0**	559	0	0	0	0	0	0	0	0	1	0
L	0	0	0	0	**11**	0	**10**	349	0	110	**14**	0
M	0	0	5	0	10	0	16	8	49	197	4	4
N	0	0	0	0	0	0	0	0	0	0	0	0
P	0	0	0	0	0	0	0	0	0	0	0	0
Q	0	0	0	**0**	0	0	0	0	0	0	0	0
R	0	438	0	0	0	0	0	0	0	74	91	0
S	0	0	0	0	0	0	0	0	0	0	0	0
T	0	0	**0**	0	0	0	0	0	0	0	0	0
V	0	0	0	0	46	0	0	**643**	0	**0**	0	3
W	0	0	109	0	0	0	640	0	0	1	890	0
Y	0	0	0	0	0	0	198	0	0	0	0	0

The table below gives the AA counts for each position. The library was constructed using five separate DNA stretches, divided by vertical bars, which contained 12 randomized positions. The counts for the native AAs are shown in bold.

**Table 4.** Problem 2 solutions: the manual library was created by an expert library designer using DCs to cover the AAs selected by Rosetta, along with the ISOR technique (33) for ensuring that the native AAs (shown in bold) were present; the library's size and error are computed as if an extra codon for the native AA (given in parentheses) were present at each position, though the ISOR technique does not guarantee that adjacent randomized positions will achieve full diversity

Solution	Pos	413	475	477	479	493	495	514	520	528	529	531	532	Totals
Manual	DCs	GCC,	ARA,	KGG,	RBA,	SNC,	KKC,	DDC,	CTG,	RYG,	CDT,	WGG,	GCC,	955^a^
		(AAA)	(GAG)	YWC,	(CAG)	(TTG)	(CAT)	WKG,	(GTG)	(GGG)	RYG,	(CTG)	(ATT)	
				(ACC)				(CTG)			(GTG)			
	AAs	A**K**	**E**KR	FGHL	AGI**Q**	ADGH	CFG**H**V	CDFGI**L**M	L**V**	A**G**M	AHLMR	**L**RW	A**I**	
				**T**WY	RTV	**L**PRV		NRSVWY		TV	T**V**			
	#NAs	2	3	7	7	9	5	14	2	5	8	3	2	8.9 × 10^7^
	#AAs	2	3	7	7	9	5	13	2	5	7	3	2	6.4 × 10^7^
	%Des	100	100	71	57	67	60	57	100	60	86	100	100	4.9
	Error	10	3	7	0	12	1	2	8	0	6	5	7	61

LibDesign	DCs	RMA	RRA	NNK	VNA	SNC	YWC	TDK	KTA	GSA	VNK	TKG	RYA	81^a^
	AAs	AEKT	EGKR	ACDEFGHIKLM	AEGIKL	ADGHL	FHLY	CFLWY	LV	AG	ADEGHIK	LW	AITV	
				NPQRSTVWY	PQRTV	PRV		*STOP*			LMNPQR			
				*STOP*							STV			
	#NAs	4	4	32	12	8	4	6	2	2	24	2	4	9.1 × 10^8^
	#AAs	4	4	21	11	8	4	6	2	2	16	2	4	3.6 × 10^8^
	%Des	50	75	34	33	63	50	67	100	100	54	100	75	0.4
	Error	10	3	0	0	12	24	149	8	49	1	96	4	356

DP Sol. 1	DCs	RMA	RRA	DBS	VDA	SNC	YWC	WDS	STA	GSA	VNS	TKG	RYA	795^a^
	AAs	AEKT	EGKR	ACFGIL	EGIK	ADGH	FHLY	CFIKLM	LV	AG	ADEGHIK	LW	AITV	
				MRSTVW	LQRV	LPRV		NRSWY			LMNPQR			
								*STOP*			STV			
	#NAs	4	4	18	9	8	4	12	2	2	24	2	4	7.6 × 10^8^
	#AAs	4	4	12	8	8	4	12	2	2	16	2	4	3.0 × 10^8^
	%Des	25	50	28	25	63	50	67	100	100	46	100	75	0.4
	Error	10	3	34	21	12	24	47	8	49	1	96	4	309

DP Sol. 2	DCs	AAA,	RRA	DSG	VNA	SNC	CAC	DKG,	STA	RBG	CWC,	WKG	RYA	966^a^
		GSA		YWC			KKC	WWC			RBG			
	AAs	AGK	EGKR	AFGHL	AEGIKL	ADGHL	CFG	FGILMN	LV	AGM	AGHLM	LM	AITV	
				RSTWY	PQRTV	PRV	HV	RVWY		RTV	RTV	RW		
	#NAs	3	4	10	12	8	5	10	2	6	8	4	4	8.9 × 10^8^
	#AAs	3	4	10	11	8	5	10	2	6	8	4	4	8.1 × 10^8^
	%Des	100	75	60	33	63	60	70	100	50	87	100	75	1.3
	Error	1	3	6	0	12	1	2	8	0	1	1	4	39

DP Sol. 3	DCs	AAA,	RVA	DBG,	ATA,	ATR,	GSA	ATR,	VTG	ATG,	DKG,	WKG	ATR,	999^a^
		GSA,		HWC	CAA,	SNC	YWC	KGG,		GSA	SMC		GYA	
		TTC			GSA			YWC						
	AAs	AFGK	AEG	AFGHILMN	AGIQ	ADGHI	AFG	FGHIL	LMV	AGM	ADGHLM	LM	AI	
			KRT	RSTVWY		LMPRV	HLY	MWY			PRVW	RW	MV	
	#NAs	4	6	15	4	10	6	8	3	3	10	4	4	1.0 × 10^9^
	#AAs	4	6	14	4	10	6	8	3	3	10	4	4	9.3 × 10^8^
	%Des	100	67	53	100	70	67	100	100	100	80	100	100	13.3
	Error	0	0	0	0	0	0	0	0	0	0	1	0	1

For the automated solutions, the library size was restricted to 10^9^ and the wild-type AA was required to be covered by the DCs themselves. The maximum achievable LibDesign score for this problem is 1000 (higher is better). The three DP solutions represent allowing one DC per position, allowing two DCs per position restricted to 10 primers total and allowing three DCs per position restricted to 25 primers total (though only 24 were chosen). See Table 2 for a description of row labels. The manual solution took hours to generate, LibDesign took 52 min 37 s and the three DP solutions took 1.40, 0.23 and 0.89 s.

In the automated solutions for this design problem, the libraries were forced to include the wild-type AA at each position, mimicking the use of the ISOR technique in the manually-constructed library. DP solutions 1 and 2 were similarly constrained as in Problem 1. DP solution 3 was constrained to use at most 25 primers. The automated solutions were limited to a 10^9^ library size, since that was the intended size for this library, even though the manual solution was an order of magnitude smaller. DP solutions 4–6, constructed with a diversity of 10^8^, are given in Table S1.

Compared against the manual solution, the DP solutions to Problem 2 were mixed. The manual solution itself included the use of multiple DCs at some positions and took advantage of the ISOR technique, which likely explains why it achieved a lower error than DP solution 1, which used only a single DC per position. DP solution 2, however, does produce a lower error than the manual solution, even though it does not use the ISOR technique. It chooses to place two DCs at the same positions that the manual solution does and two more in the other two stretches that the manual-library's designer chose not to explore. As a result, it achieves a slightly larger AA diversity, though, at the cost of almost 10× higher DNA diversity. Now, we have calculated the AA diversity for the manual library by treating the wild-type AA—which the ISOR technique adds—as if it were encoded by a second (or third) DC at each position. This overestimates the manual library's diversity, since if one position receives its wild-type AA, then its adjacent positions will also receive their wild-type AAs. In spite of this overestimation, DP solution 2 exceeds the manual library's AA diversity.

DP solution 3 reduces the error still further. It achieves an error of 1, missing only the lysine at position 531 that appeared once in the Rosetta simulations. It also achieves a 14:15 AA:DNA ratio, coding for a single AA at position 377 with two codons and all others at all positions with only a single codon. DP solution 3 included several AAs that were not observed in the Rosetta designs; we cannot conclude, then, that the use of multiple DCs is perfectly molding the DNA to the desired set of AAs. The error function offers no penalty for including unobserved AAs (simply no bonus for doing so), so the DP algorithm's inclusion of extra unobserved AAs in attempting to cover all the observed AAs is expected. However, the high AA:DNA ratio is not expected since this ratio is not directly optimized. The unobserved AAs can be squeezed out if the diversity limit is dropped, at the expense of increasing the resulting error. A seventh DP solution (Table S1) which was constrained in the same ways that DP solution 3 was, except that its diversity limit was 10^7^, defines a library with an error of 69—similar to that produced by the manual solution—and which contains only six AAs that were unobserved in the original designs. DP solution 7 achieves a perfect 1:1 AA:DNA ratio.

For problem 2, the DP solutions were completed much more quickly than the manual one. The manual solution took many hours to construct, whereas DP solutions 1, 2 and 3 took 1.40, 0.23 and 0.89 s.

In both problems, the DP solutions achieved lower error levels than the LibDesign solutions, which is not unexpected given that LibDesign was not optimizing our error metric. What was unexpected, however, was that the DP solutions as well as the manual solutions achieved a better (higher) LibDesign score than LibDesign did. Since LibDesign relies on exhaustive enumeration of the codons it chose at the beginning, this result suggests that the original choices for which codons to consider were sub-optimal. The ability of the dynamic-programming libraries to achieve such high LibDesign scores (the highest possible score for Problem 1 was 200, the highest possible score for Problem 2 was 1000; the third DP solutions achieved scores of 192 and 999) is the result of having achieved such low errors; almost all of the AAs that came out of the computational designs were covered, resulting in very high LibDesign scores. It is likely that it would have produced much worse LibDesign scores if its best solution had a much higher error. LibDesign's reliance on brute force enumeration meant that it took much longer to produce its libraries than the DP algorithm; at Problem 1, it took 18 min and 51 s, at Problem 2, it took 52 min 37 s.

The Supplemental Material presents the results from an additional 798 library design problems from 38 proteins where we selected subsets of residues of varying sizes and redesigned those residues with Rosetta. We report the error level (Table S3) and running time (Table S4) as we varied (i) the diversity limit, (ii) the number of DCs per position and (iii) the primer limit. These results show that if one started from a DC library using only a single DC per position and had the choice between either making the library 10× larger or allowing two DCs per position and 10 primers total, that the error reduction is much greater for the latter rather than the former (23% reduction versus 75% reduction; *P*-value < 0.00001, 1-tailed *t*-test). The median running time for these 798 jobs was 0.61 s, with the longest job taking 104 s.

### Comparison with ILP

This section concludes with a comparison between our DP algorithm and the previously-published ILP formulations of the problem. ILP, sometimes called mixed integer programming, is an NP-Complete problem (34) where many problem instances admit a rapid solution. Indeed, the frequency with which ILP problems present themselves in process optimization has led to a wide availability of commercial ILP solvers, including the free GLPK solver, used in Chen *et al*. (28) and which we have used here. When an ILP solver produces a solution, which is not guaranteed, it produces the exact solution and so it is a natural comparison point for our DP algorithm which also produces an exact solution.

In the Supplemental Materials, we give a reformulation of the previously published ILP solutions for a single DC per position that optimizes our error metric and a novel ILP formulation to allow multiple DCs per position. The multiple DC formulation requires computing the sum of a product of variables, which is challenging to do with ILP and so it runs slowly.

There are four principle advantages that DP has over ILP: DP runs in polynomial time whereas ILP is NP-complete, DP is faster, a single DP execution can provide more than a single solution and DP is more easily implemented than ILP. To show that DP is faster, we compared ILPs running time against DP's running time for the two problems presented above and in the Supplemental Materials for 268 additional jobs. The running times for problems 1 and 2 are given in Table 5. In five of the six cases, DP completed in less time than ILP. Library design jobs where multiple DCs were allowed degraded the ILP running time substantially. For 192 of the 220 jobs using multiple DCs from the Supplemental Materials, DP completed in less time than ILP. In 107 of those cases, no ILP solution was obtained in 1000 min at which point we killed the ILP solver. Assuming these jobs had finished in exactly 1000 min, the median speedup for DP over ILP when using multiple DCs was 5752.

**Table 5.** DP and ILP running times (s) for the three variations on the two library design problems

	Prob 1	Prob 1	Prob 1	Prob 2	Prob 2	Prob 2
	1 DC	2 DCs	3 DCs	1 DC	2 DCs	3 DCs
DP	0.15	0.28	13.57	1.40	0.23	0.89
ILP	0.46	661.08	1607.67	0.09	46.86	4.09

The running times for the ILP solutions include only the amount of time for the *glpsol* solver to run, and do not include the preprocessing step of examining DC combinations; the running times for the DP solutions include both preprocessing and optimization steps. Running times were measured on a 2013 MacBookPro with a 2.3 GHz i7 processor and 4 GB of RAM. DP was run within Chrome version 34.0.1847.13.

A single execution of DP produces more than just a single library. The DP algorithm computes the size of the smallest library capable of achieving every error level starting at 0 and building upward until it finds a library that is beneath the given size limit. As a result, it can report each of those library sizes as a function of error level to give the user insight into how the error changes as a function of library size. Without having to re-run the algorithm, they can see what would happen to the error as they increased the size of their library. Furthermore, DP can provide the codons that comprise the larger libraries if the user is interested in them in O(*n*) time. SwiftLib presents users with a scatter plot of the Pareto-optimal libraries—scored by library size and error level—with the log-library size given on the x-axis and the error level on the y-axis. They can then click on any of the dots in the plot to display the codons that make up the library that it corresponds to.

The fourth advantage, that DP has a simpler implementation than ILP, means that we are able to create a website that has no back end. This site simply serves up the code to run DP (which can be downloaded from the website or from github: https://github.com/aleaverfay/swiftlib_javascript) and the code is executed within the user's web browser; the heavy computation is performed on the user's own computer. In contrast, a website that relied on an ILP solution would have to queue ILP jobs to be performed on a back end server and then delivered results as they completed. Even if ILP were as fast as DP, such a server might accumulate a heavy backlog of work. SwiftLib, needing only to deliver the DP source code, could accommodate many more users before it started to slow down.

The largest drawback of DP in comparison to ILP is in its restricted objective function. With ILP, one is able to formulate more complicated functions to optimize, such as the pairwise quality and novelty metrics presented by Parker *et al*. (27). It it our opinion, however, that the advantage of being able to consider multiple DCs per position (within a reasonable amount of time) outweighs the weakness in not being able to incorporate pairwise data.

## DISCUSSION

Directed evolution is rapidly becoming a standard complement to computational protein design (13,24,26,28,35–46). It allows protein designers to test vastly more designs than could individually be expressed, purified and assayed. As a result, shortcomings in the current generation of energy functions and sampling protocols can be overcome, and useful proteins that vary only slightly in their sequences from a starting design can be found. Since there are so many ways for a design to fail, some of the best insight can come from finding a successful design and contrasting it against the sequences that the design score function most favors. Directed evolution offers a mean to find such successful designs.

DC libraries are a natural complement to computational protein design as they allow a designer to focus diversity to the active site or interface positions in ways that error-prone PCR, for example, could not. However, DC libraries are quite difficult to optimize by hand; there are thousands of possible DCs and so finding the best one that also offers a reasonable compromise with the other positions being optimized while the whole library stays beneath a given diversity limit is a daunting task at best. The task becomes decidedly harder once the possibility of choosing multiple DCs at a single position is introduced. Moreover, manually designing DC libraries is highly error prone, as observed in this study. It is no surprise that automated construction of DC libraries has been the focus of several prior studies (23–24,26–28). SwiftLib offers a rapid solution to the design of DC libraries. Because it expects as inputs a set of AA counts for each position to be randomized, which are readily derived from the outputs of protein design simulations, it should fit naturally into the computational-protein designer's workflow.

SwiftLib was able to find libraries that covered nearly every AA present in the input designs when it was allowed to consider multiple DCs at each position. SwiftLib could make it much easier to screen libraries that cover the potentially-useful AAs as suggested by computational design or multiple-sequence alignments by reducing the number of sequences that have to be tested to achieve full coverage.

## SUPPLEMENTARY DATA

Supplementary data are available at NAR Online.

SUPPLEMENTARY DATA
