# Supplementary material for: SwiftLib: rapid degenerate-codon-library optimization through dynamic programming
Source: Nucleic Acids Res. 2014 Dec 24;43(5):e34. doi: 10.1093/nar/gku1323 (PMC4357694; doi:10.1093/nar/gku1323)
Supplement: SUPPLEMENTARY DATA [file supp_43_5_e34__index.html]

SwiftLib: rapid degenerate-codon-library optimization through dynamic programming — SUPPLEMENTARY DATA 

# SwiftLib: rapid degenerate-codon-library optimization through dynamic programming

## SUPPLEMENTARY DATA

**Files in this Data Supplement:**

- SUPPLEMENTARY DATA
- SUPPLEMENTARY DATA
